# Cerebral and Somatic Oxygen Saturation in Neonates with Congenital Heart Disease before Surgery

**DOI:** 10.3390/jcm10112455

**Published:** 2021-06-01

**Authors:** Mi Jin Kim, Jae Suk Baek, Jung A Kim, Seul Gi Cha, Jeong Jin Yu

**Affiliations:** 1 Division of Pediatric Cardiology, Department of pediatrics, Asan Medical Center, University of Ulsan College of Medicine, Seoul 05505, Korea; atlranta83@hanmail.net (M.J.K.); siminhuhu@hanmail.net (S.G.C.); jjyu@amc.seoul.kr (J.J.Y.); 2 Department of Nursing, Asan Medical Center, Seoul 05505, Korea; 1224wjddk@naver.com

**Keywords:** cerebral oxygen saturation, somatic oxygen saturation, congenital heart disease

## Abstract

Background: We investigated preoperative cerebral (ScO_2_) and abdominal (StO_2_) regional oxygen saturations according to cardiac diagnosis in neonates with critical CHD, their time trends, and the clinical and biochemical parameters associated with them. Methods: Thirty-seven neonates with a prenatal diagnosis of CHD were included. ScO_2_ and StO_2_ values were continuously evaluated using near-infrared spectroscopy. Measurements were obtained hourly before surgery. A linear mixed effects model was used to assess the effects of time and cardiac diagnosis on regional oxygenation and to explore the contributing factors. Results: Regional oxygenation differed according to cardiac diagnosis (*p* < 0.001). ScO_2_ was lowest in the patients with severe atrioventricular valvar regurgitation (AVVR) (48.1 ± 8.0%). StO_2_ tended to be lower than ScO_2_, and both worsened gradually during the period between birth and surgery. There was also a significant interaction between cardiac diagnosis and time. The factors related to ScO_2_ were hemoglobin and arterial saturation, whereas no factor was associated with StO_2_. Conclusions: Preoperative ScO_2_ and StO_2_ in critical CHD differed according to cardiac diagnosis. ScO_2_ in the patients with severe AVVR was very low, which may imply cerebral hypoxia. ScO_2_ gradually decreased, suggesting that the longer the time to surgery, the higher the risk of hypoxic brain injury.

## 1. Introduction

Neurobehavioral impairments are common in infants undergoing surgery for CHD, and they occur across a wide spectrum; cognition, motor, social interaction and behavior, language, inattention, and executive function [1,2,3,4,5,6,7,8,9,10]. Many studies have discussed the factors related to surgery or postoperative care on this issue. However, some studies suggested that brain damage may occur during the early postnatal life of infants with CHD, which may lead to impaired neurodevelopment [11,12].

Especially in patients with complex CHD, these abnormalities will be exacerbated by unstable hemodynamics before undergoing cardiac surgery. Therefore, maintaining stable hemodynamics before cardiac surgery and recognizing clinical deterioration early and taking appropriate action will yield a better outcome. Therefore, it is necessary to have an in-depth understanding of cerebral perfusion and its relationship to underlying heart diseases. Moreover, the deterioration of other-end organ perfusion, which may affect the proper timing of surgery or the outcome of the surgery, should be monitored.

Near-infrared spectroscopy (NIRS) is a tool used to continuously and non-invasively monitor the degree of oxygenation of multisite regional tissues, such as the brain, intestines, and kidneys. There have been many studies on NIRS monitoring of infants with CHD during or after surgery; however, there have been few on the preoperative cerebral and somatic oxygenation in the neonates with CHD.

The objective of this study was to investigate preoperative cerebral (ScO_2_) and somatic (StO_2_) oxygenation according to cardiac diagnosis using NIRS and the time course of these parameters. We also investigated the extent of changes in ScO_2_ and StO_2_ at the time of clinical deterioration. Moreover, we explored the clinical and biochemical parameters that correlate with ScO_2_ and StO_2_.

## 2. Patients and Methods

### 2.1. Patients

A prospective study was performed at a tertiary cardiac and neonatal intensive care unit (NICU) of Asan medical center. The study was approved by the institutional review board of Asan medical center (IRB number 2018-0817) and written informed consent was obtained.

All neonates with a prenatal diagnosis of congenital heart disease who were born at a gestational age >36 weeks between September 2018 and August 2019 were considered for inclusion. Patients with other congenital anomalies or those who were transferred to another hospital before surgery were excluded.

The study population was divided according to cardiac diagnosis:

Group 1: normal heart

Group 2: transposition of the great arteries (TGA)

Group 3: duct-dependent systemic circulation; Coarctation of aorta (CoA) with ventricular septal defect (VSD), interrupted aortic arch (IAA) with VSD, functional single ventricle (FSV) with CoA

Group 4: duct-dependent pulmonary circulation; tetralogy of fallot (TOF) with pulmonary atresia (PA) or pulmonary stenosis (PS), FSV with PA or PS

Group 5: regurgitation lesion; tricuspid dysplasia, Ebstein’s anomaly

Group 6: mixing lesion; truncus arteriosus, total anomalous pulmonary venous return (TAPVR)

### 2.2. Regional Oximetry

We used INVOS™ 5100c near-infrared spectrometers (Somanetics, Troy, MI, USA) with neonatal somasensors. The somasensors were placed on the two sites (forehead and abdomen) to record ScO_2_ and StO_2_ oximetries, and they were measured continuously during their stay in the NICU. The cerebral sensor was placed on the bilateral forehead and the somatic sensor was placed on the anterior abdomen (just below the umbilicus) [13]. ScO_2_ and StO_2_ were continuously monitored and recorded by the device every 6 s. For analysis, measurements were acquired hourly and at specific time points whenever an adverse event occurred.

### 2.3. Data Collection

We collected all available clinical and biochemical parameters that may have influenced the course of ScO_2_ and StO_2._ We reviewed the electronic medical records of the study cohort for the collection and collation of dates regarding patients’ baseline characteristics, underlying heart disease and clinical course, gestational age, birth weight, Apgar score at 5 min, respiratory support, and treatment with inotropes and sedatives.

We also measured serum lactate, hemoglobin, and hematocrit from the venous blood samples; pH, pCO_2_, and pO_2_ from the arterial, venous, or capillary blood gas measurement, as well as arterial saturation (SpO_2_), blood pressure, heart rate, and body temperature. Blood gas measurement was conducted upon admission to the NICU and every 6 to 12 h during the preoperative period. SpO_2_ and heart rate were continuously monitored. Blood pressure was measured continuously using an arterial catheter or every hour using a NIBP monitor. Body temperature was measured every 4 to 6 h. For analysis, measurement data were sampled on an hourly basis for continuous monitoring of parameters while all measurement data were used for intermittent monitoring of parameters.

### 2.4. Statistical Analysis

For statistical analysis, we used SPSS 21.0 (IBM Corp., Armonk, NY, USA). Continuous variables were summarized using standard descriptive statistics (mean and standard deviation), and frequencies and percentages were used for categorical variables.

We displayed the course of ScO_2_ and StO_2_ graphically per hour according to cardiac diagnosis. To analyze differences in ScO_2_ and StO_2_ according to cardiac diagnosis, we used the Wilcoxon signed rank test. The preoperative time trends in ScO_2_ and StO_2_ were examined using a linear mixed model.

We used the Pearson’s correlation test to determine the correlation between demographic and physiological parameters and regional oxygen saturation. To explore the factors contributing to regional oxygenation, a linear mixed model including a potential subset of predictors was used. The variable selection was performed using the Akaike information criterion. *p*-values of <0.05 were considered to be statistically significant.

## 3. Results

### 3.1. Patient Characteristics

A total of 37 neonates were included in the study. Reasons for exclusion were transfer or expiration within 72 h after birth in 2 patients, major chromosomal abnormalities in one patient and consent withdrawal after enrollment in 2 patients. Patient characteristics are presented in Table 1. The thirty-seven neonates had a gestational age of 38.8 ± 1.1 weeks, birth weight of 3.2 ± 0.5 Kg and birth height of 49.4 ± 2.6 cm. Patients were classified according to their postnatal diagnosis: normal heart (*n* = 3), TGA (*n* = 4), duct-dependent systemic circulation (*n* = 10), duct-dependent pulmonary circulation (*n* = 13), severe atrioventricular valvar regurgitation (AVVR) (*n* = 2), total anomalous pulmonary vein return (TAPVR) or Truncus arteriosus (*n* = 5).

### 3.2. Cerebral and Somatic Oxygenation

**Figure 1:**
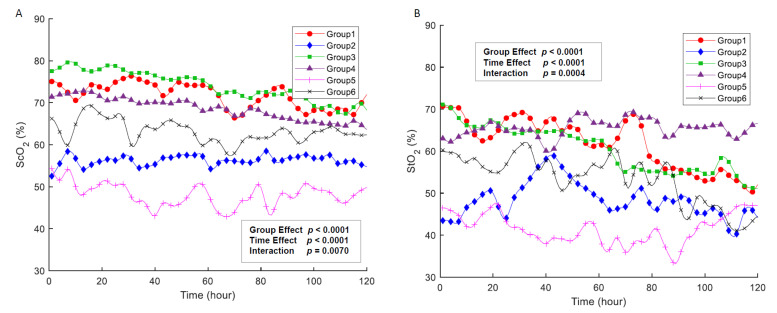
Cerebral (**A**) and somatic (**B**) oxygen saturation over the 120 h after birth. ScO_2_ and StO_2_ decreased gradually with time (*p* = 0.029) and differed according to cardiac diagnosis (*p* < 0.001). There was also a significant interaction between cardiac diagnosis and time (*p* = 0.007). ScO_2_; cerebral oxygenation, StO_2_; Somatic oxygenation. Group 1; normal heart, Group 2; transposition of the great arteries, Group 3; duct-dependent systemic circulation, Group 4; duct-dependent pulmonary circulation, Group 5; regurgitation, Group 6; mixing lesion.

Table 2 and Figure 1 show the ScO_2_ and StO_2_ values according to the underlying heart disease. In Figure 1, we smoothed the raw data samples with a Gaussian-weighted moving average filter, using a window of length 10. The regional oxygenation was different according to cardiac diagnosis. ScO_2_ significantly decreased in the patients with severe AVVR (48.1 ± 8.0%), TGA (56.3 ± 11.3%), mixing lesions (63.2 ± 10.6%), and CHD with severe PS or PA (68.7 ± 7.5%). ScO_2_ values were similar between patients with aortic coarctation or interrupted aortic arch (74.8 ± 7.9%) and those with normal hearts (71.7 ± 10.3%).

StO_2_ was lower on average than ScO_2_ in all groups except in the regurgitation lesion group. For cardiac diagnosis, we observed the lowest StO_2_ in the regurgitation lesion group and the highest in the duct-dependent systemic circulation group. The second lowest StO_2_ was observed in the TGA group, followed by the mixing lesion, duct-dependent systemic circulation, and normal heart groups (Table 2 and Figure 1).

**Figure 2:**
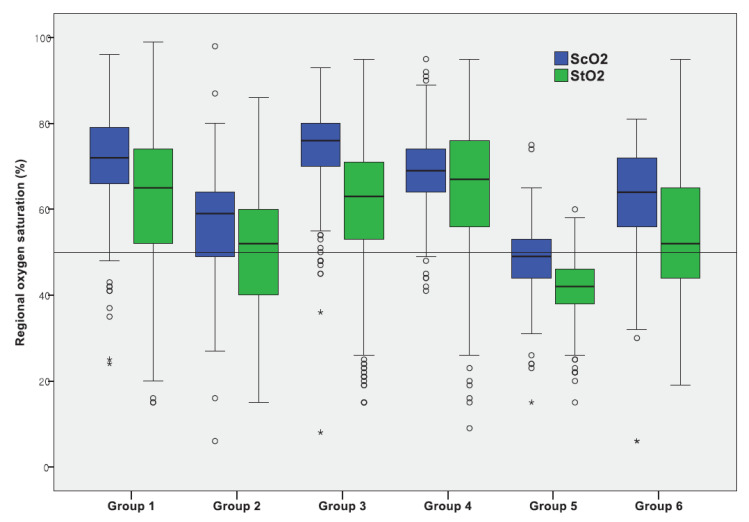
Cerebral and somatic oxygen saturation according to underlying heart disease. Circles indicate outliers, asterisks are the extreme outliers. ScO_2_; cerebral oxygenation, StO_2_; Somatic oxygenation. Group 1; normal heart, Group 2; transposition of the great arteries, Group 3; duct-dependent systemic circulation, Group 4; duct-dependent pulmonary circulation, Group 5; regurgitation, Group 6; mixing lesion.

We used a linear mixed effects model to assess the effects of time and cardiac diagnosis on ScO_2_ and StO_2_ (Figure 2, Table 3). Figure 2 shows the preoperative temporal trend of regional oxygen saturation in each group over the 120 h after birth. ScO_2_ and StO_2_ decreased gradually with time (*p* = 0.029), and they differed according to cardiac diagnosis (*p* < 0.001). There was also a significant interaction between cardiac diagnosis and time (*p* = 0.007); ScO_2_ decreased significantly with time in Groups 3 and 4.

### 3.3. Association with Regional NIRS and Physiologic Parameters

Supplementary Table S1 shows the correlation between regional oxygen saturation (ScO_2_, StO_2_) and the clinical variables. Arterial saturation and hemoglobin concentration showed weak positive correlation with ScO_2_ and StO_2_, while PaCO_2_ showed weak negative correlation. However, blood pressure was not correlated with regional oxygenation.

We used a linear mixed model, including potential subsets of predictors, to explore the factors contributing to regional oxygenation. The factors related to ScO_2_ were hemoglobin (*p* = 0.015) or hematocrit (*p* = 0.019) and arterial saturation (*p* = 0.006), whereas there was no factor related to StO_2_.

The serum lactate had weak negative correlation with cerebral oxygenation (Supplementary Figure S1) (ScO_2_; r = −0.218, *p* < 0.001) and no correlation with somatic oxygenation.

### 3.4. Changes in Regional Oxygenation in Patients with Adverse Events

There were serious adverse events in six patients; 1 recurrent paroxysmal supraventricular tachycardia, 1 focal seizure, 2 cases of sepsis, 1 liver hematoma, and 1 hematochezia suspected of necrotizing enterocolitis (NEC). Patients with adverse events had abrupt changes in regional oxygenation during NIRS monitoring before symptoms such as seizure, fever, hematochezia, and lethargic appearance occurred. Baseline cerebral and somatic oxygenation remained stable and suddenly decreased approximately 40 min before the adverse events occurred (Supplementary Figure S2). This rapid decrease occurred in both cerebral and somatic regional oxygenation in the patients with sepsis or seizures, whereas the decrease occurred dominantly in somatic oxygenation in the patient with NEC.

Change in regional oxygenation also occurred after adequate management; in patients with TGA with intact ventricular septum (IVS) and restrictive atrial mixing, who showed very low values of ScO_2_ and StO_2_ shortly after birth, ScO_2_ and StO_2_ improved after emergency atrial septostomy.

## 4. Discussion

Neurobehavioral impairments are common in infants with CHD and occur across a wide spectrum [1,2,3,4,5,6,7,8,9,10]. The etiological factors of these problems are multifactorial and comprise a complex interaction between several factors before, during, and after surgery.

As preoperative factors, chronic hypoxia, acidosis, poor nutrition, and inadequate cerebral perfusion due to hemodynamic instability are the possible factors contributing to brain injury. Our study shows that cerebral oxygenation before surgery is significantly low in patients with CHD and very low in a few patients with certain CHD, which may imply cerebral hypoxia.

A few studies have reported differences in preoperative regional oxygenation in various types of CHD [14,15,16]. However, these studies only measured regional oxygenation for a very short preoperative period while focusing mainly on changes after the operative period [14,16]. Our study showed that there was a difference in regional oxygenation depending on cardiac diagnosis, even though the difference was not large in arterial oxygen saturation as measured by SpO_2_, and that the value of regional oxygenation was significantly lower than that in the normal heart group. The patients with severe tricuspid regurgitation (TR) (Group 5) and the patients with TGA with IVS (Group 2) had the lowest value of regional oxygenation, which is aligned with general expectation. It can be assumed that Group 5 faced the most severe low cardiac output state. During the immediate postnatal period, while pulmonary vascular resistance is still elevated, severe TR results in massive cardiomegaly and heart failure. In addition, it may be assumed that Group 2 showed a lower value due to limited effective mixing and a relatively low arterial saturation.

Normative ScO_2_ values have been reported as 76.8% ± 8.5% in healthy neonates. ScO_2_ value in many children with CHD is less than that in healthy children. In critical CHD, ScO_2_ was far lower than normal; mean values for ScO_2_ were as follows: 46.8 ± 8.9% in TGA, in hypoplastic left heart syndrome, 52.0 ± 7.2% [17], in PA, 38 ± 6%, and in TOF, 57 ± 12% [14]. Our study also showed that, overall, ScO_2_ in CHD patients was lower than normal. Contrary to previous reports [14,17], the value of ScO_2_ in our study was not significantly different to the normal values, with decreased pulmonary flow in right to left shunt lesion patients and higher than normal in left obstructive lesion patients, although our study did not include neonates with hypoplastic left heart syndrome. This can be attributed to appropriate and timely management for these patients; they were admitted to the neonatal intensive care unit (NICU) shortly after birth and prostaglandin E_1_ infusion was continued throughout the preoperative period to maintain ductal patency immediately after diagnosis. In addition, mechanical ventilator support and adequate inotropic helped maintain hemodynamic stability.

In our study, patients with severe AVVR, such as Ebstein’s anomaly or tricuspid valve dysplasia, showed the lowest value of ScO_2_ as 48.1 ± 8.0%.

Although there are no human pediatric data, Kurth et al.’s study in neonatal piglets showed that cerebral functional impairment begins at ScO2 of about 45%; there is a buffer zone between 45% and 60% whereby cerebral oxygenation is adequate for function but lower than normal. Depending on the result of that study, the value of ScO_2_ in the severe AVVR group is in a buffer zone. This means that the group is very susceptible to neurodevelopmental complication. In addition, ScO_2_ gradually decreased with time, which was similar to the results of the Lynch JM et al. Study [17,18], suggesting that the longer the time between diagnosis and surgery, the higher the risk of hypoxic brain injury. In particular, Groups 3 and 4 had a decreasing trend in ScO_2_ during the preoperative period. In both groups, with ductal dependent congenital heart disease, unbalanced Qp/Qs, insufficient ductal patency, or both may have resulted in such a trend.

Somatic oxygenation was lower than cerebral oxygenation in this study. In other words, this finding means that the somatic–cerebral difference is negative. The somatic–cerebral difference was 10–20% in healthy patients and decreased to 0% or negative values when in a low cardiac output state [19]. Some studies reported that a somatic–cerebral saturation difference was associated with mortality during the early postoperative period [19,20]. Our findings may imply that many patients with critical CHD are confronted with low cardiac outputs despite appropriate medical treatment. In particular, patients with severe TR during the transient circulation period are faced with severe impairment in both ScO_2_ and StO_2_.

The interest related to preoperative StO_2_ monitoring in the CHD is because it may be useful to predict adverse events caused by the impairment of systemic perfusion, such as acute kidney injury and NEC, which can affect the pre/post-operative course and outcome. Adverse events commonly occur in CHD patients [5,6,21], and these may originate pre, during, or post-surgery, as a result of global ischemia and hypoxia.

In our study, preoperative adverse events occurred in 6 patients (16.2%) including sepsis (*n* = 2), seizure (*n* = 1), abdominal problems (*n* = 2), and arrhythmia (*n* = 1). The value of StO_2_ in these patients declined abruptly in relation to an adverse event. In particular, it decreased before the onset of clinical symptoms in 2 neonates with NEC and in 1 neonate with sepsis.

However, it is difficult to define a critical threshold of adverse events. Absolute normative abdominal StO_2_ values do not seem to be clear. Abdominal (infraumbilical) StO_2_ ranged widely from 32% to 66% in healthy newborns and preterm infants, whereas renal StO_2_ was reported as 86.8% ± 8.1% with a range of 64% to 7% [22,23].

This difference is caused by the motility and varying luminal contents of the small bowel [24]. Therefore, the clinical utility of abdominal StO_2_ lies in trend monitoring rather than in critical threshold values. This enables early recognition of clinical deterioration based on the change in value from baseline. A few previous studies reported that monitoring through NIRS in pediatric intensive care units after cardiac surgery enables early prediction of serious adverse events [25,26]. Mebius et al. reported that cerebral and/or renal regional oxygenation changed 30 min or more before sudden, unexpected clinical deterioration, while other hemodynamic variables did not indicate that this deterioration was imminent.

Therefore, there remains a question of how much change in value should be considered significant. McNeill et al. reported that regional oxygenation fell by 30–35% relative to the baseline before the loss of output [15,23]. Fluctuations in regional oxygenation exceeding a reduction of 30–35% in one individual indicate the potential for decreased end-organ perfusion and consequent deterioration in clinical status. Changes in regional oxygenation using NIRS monitoring should be considered as a red flag and should be fully screened in infants, alongside physical examination, abdominal or brain ultrasound imaging study, and basic blood tests.

In this study, the factors contributing to ScO_2_ were hemoglobin (*p* = 0.015) or hematocrit (*p* = 0.019) and arterial saturation (*p* = 0.006), whereas no factor was related to StO_2_. This finding may indicate that certain strategies might decrease the risk of cerebral hypoxic-ischemic injury before surgery. Blood pressure was not a factor contributing to regional oxygenation; we suppose that it was kept in the cerebral autoregulation range.

Previous studies showed conflicting results regarding the association between serum lactate and cerebral near-infrared spectroscopy in infants after cardiac surgery. One study showed that an average cerebral and renal regional saturation of less than 65% as measured by NIRS predicts hyperlactatemia (>3 mmol/L) in acyanotic children after congenital heart surgery [27]. Meanwhile, other studies reported no correlation between cerebral regional saturation and serum lactate during or after cardiac surgery [28,29]. Our study showed a very weak correlation between regional oxygenation on NIRS and serum lactate. This may be because, even if global hypoperfusion occurs to the extent that lactate increases, cerebral oxygenation is maintained to some extent.

Our study is limited because it was conducted in a single center, with a small number of infants, over a short period of time, and it has location sampling bias. Additionally, poor performance may be related to the position of the NIRS probe. Increasing patient population with focused grouping remains as future work.

This study showed that preoperative ScO_2_ in critical CHD patients was relatively low and differed according to cardiac diagnosis. In particular, ScO_2_ in the patients with severe AVVR was very low, which may imply cerebral hypoxia. Abdominal StO_2_ tended to be lower than ScO_2_, and both values worsened gradually during the peri-operative periods; this finding suggests that the longer the time between birth and surgery, the higher the risk of hypoxic brain injury.

In addition, we showed that abrupt changes in regional oxygenation occurred in relation to adverse events. We believe that the continuous monitoring of regional oxygenation helps to maintain stable hemodynamics before the cardiac surgery, recognize clinical deterioration early, and take appropriate action.

## Figures and Tables

**Table 1:** Subject demographics.

Variable	Value (*n* = 37)
Gestational age, weeks	38.8 ± 1.1
Birth weight, Kg	3.2 ± 0.5
Birth height (cm)	49.4 ± 2.6
Male, *n* (%)	20 (54)
Apgar 5 min	8.4 ± 1.0
Diagnosis, *n* (%)	
Duct-dependent pulmonary circulation (TOF with PA or PS, FSV with PA or PS)	11 (29.7)
TGA with IVS	4 (8.1)
Mixed lesion (Truncus arteriosus, TAPVR)	8 (21.6)
Duct-dependent systemic circulation (CoA with VSD, IAA with VSD, FSV with CoA)	11 (29.7)
Severe valve regurgitation (TV dysplasia, Ebstein’s anomaly)	2 (5.4)
Normal heart	3 (5.4)
Respiratory support, *n* (%)	25 (67.5)
Length of stay in the NICU, days	12.9 ± 10.7

All values are presented as mean ± SD, unless stated otherwise. CoA, coarctation of the aorta; IVS, intact ventricular septum; FSV, functional single ventricle; TAPVR, total anomalous pulmonary venous return; PA, pulmonary atresia; PS, pulmonary stenosis; TOF, tetralogy of fallot; TGA, transposition of the great arteries.

**Table 2:** SpO_2_ and cerebral and somatic tissue oxygenation measurement.

	Group 1 (N = 3, *n* = 479) Mean ± SD	Group 2 (N = 4, *n* = 466) Mean ± SD	Group 3 (N = 10, *n* = 1173) Mean ± SD	Group 4 (N = 13, *n* = 1151) Mean ± SD	Group 5 (N = 2, *n* = 198) Mean ± SD	Group 6 (N = 5, *n* = 253) Mean ± SD	*p*-Value
SpO_2_ (%)	92.8 ± 4.8 ^a,d^	84.5 ± 7.3 ^b^	93.5 ±5.8 ^d^	91.8± 7.3 ^a^	87.9± 6.9 ^c^	93.0± 4.0 ^d^	0.000
Cerebral oximetry (ScO_2_) (%)	71.7 ± 10.3 ^a^	56.3 ±11.3 ^b^	74.8± 7.9 ^c^	68.7± 7.5 ^d^	48.1± 8.0 ^e^	63.2 ± 10.6 ^f^	0.000
Somatic oximetry (StO_2_) (%)	61.7 ± 16.2 ^a^	48.6 ±15.8 ^b^	61.7 ± 13.8 ^a^	65.4± 14.2 ^c^	51.3± 7.5 ^d^	53.7± 13.9 ^e^	0.000
*p*-value (between ScO_2_ and StO_2_)	0.000	0.000	0.000	0.000	0.715	0.000	

N: number of patients, *n*: number of measurement samples. ^a^, ^b^, ^c^, ^d^, ^e^ and ^f^ represent the difference between groups, the same letters indicate non-significant differences among groups, while the different letters indicate the group differs significantly from others. Group 1: normal heart, Group 2: transposition of the great arteries, Group 3: duct-dependent systemic circulation, Group 4: duct-dependent pulmonary circulation, Group 5: regurgitation lesion, Group 6: mixing lesion.

**Table 3:** Parameter estimates of linear mixed model.

		ScO_2_			StO_2_		
Effect		Estimate	SE	*p*-Value	Estimate	SE	*p*-Value
Intercept		74.917	1.648	<0.0001	70.962	2.961	<0.0001
Time point		−0.051	0.023	0.0290	−0.150	0.042	0.0004
Group	1	0.000			0.000		
	2	−19.490	2.343	<0.0001	−21.411	4.209	<0.0001
	3	4.937	1.921	0.0106	−1.273	3.451	0.7125
	4	−1.985	1.937	0.3061	−6.230	3.480	0.0743
	5	−24.653	2.934	<0.0001	−28.278	5.268	<0.0001
	6	−9.793	2.678	0.0003	−9.601	4.799	0.0463
Time point X Group	1	0.000			0.000		
	2	0.064	0.034	0.0606	0.130	0.060	0.0328
	3	−0.045	0.028	0.1093	0.000	0.051	0.9939
	4	−0.021	0.028	0.4460	0.161	0.050	0.0013
	5	0.014	0.046	0.7598	0.122	0.082	0.1374
	6	0.019	0.039	0.6202	0.017	0.070	0.8018
	1	0.000			0.000		

Group 1: normal heart, Group 2: transposition of the great arteries, Group 3: duct-dependent systemic circulation, Group 4: duct-dependent pulmonary circulation, Group 5: regurgitation lesion, Group 6: mixing lesion.

## Data Availability

Data is contained within the article or Supplementary Materials.

## Supplementary Materials

The following are available online at https://www.mdpi.com/article/10.3390/jcm10112455/s1, Figure S1: Relationship between averaged regional NIRS and lactate, Figure S2: Changes in the cerebral and somatic oxygenation associated with adverse events, Table S1: Correlations with regional O2 saturation.

## Glossary

AVVR	Atrioventricular valvar regurgitation
CHD	Congenital heart disease
CoA	Coarctation of aorta
FSV	Functional single ventricle
IAA	Interrupted aortic arch
NEC	Necrotizing enterocolitis
NICU	Neonatal intensive care unit
NIRS	Near-infrared spectroscopy
PA	Pulmonary atresia
PS	Pulmonary stenosis
Qp/Qs	Ratio of pulmonary-to-systemic flow
ScO_2_	Cerebral oxygenation
StO_2_	Somatic oxygenation
TAPVR	Total anomalous pulmonary venous return
TGA	Transposition of the great arteries
VSD	Ventricular septal defect
