# Supplementary material for: Cerebral and Somatic Oxygen Saturation in Neonates with Congenital Heart Disease before Surgery
Source: J Clin Med. 2021 Jun 1;10(11):2455. doi: 10.3390/jcm10112455 (PMC8199521; doi:10.3390/jcm10112455)

Journal of
Clinical Medicine

MDPI

SUPPLEMENTARY MATERIAL

Table S1. Correlations with regional O_2 saturation.

	Cerebral O_2		Somatic O_2	
**Variable**	**R**	**P-value**	**R**	**P-value**
Arterial saturation	0.449	<0.000	0.29	<0.000
Hemoglobin concentration	0.284	<0.000	0.323	<0.000
Systolic blood pressure	-0.07	<0.000	0.03	0.039
Diastolic blood pressure	0.021	0.139	0.059	<0.000
Mean blood pressure	-0.023	0.114	0.049	0.001
pH	0.033	0.329	0.024	0.479
PCO2	-0.188	<0.000	-0.378	<0.000
PO2	0.298	<0.000	0.203	<0.000
Sex	0.007	0.557	0.213	<0.000

PCO_2: partial pressure of carbon dioxide, PO_2: partial pressure of oxygen.

Figure S1. Relationship between averaged regional NIRS and lactate.

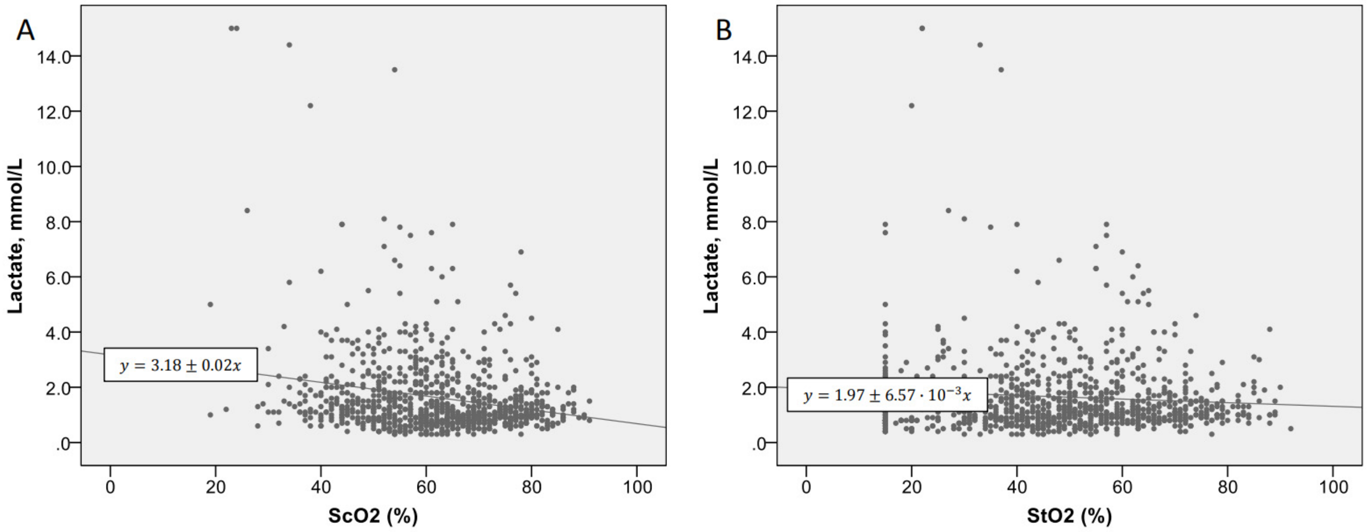

ScO_2; cerebral oxygenation, StO_2; Somatic oxygenation

Figure S2. Changes in the cerebral and somatic oxygenation associated with adverse events.

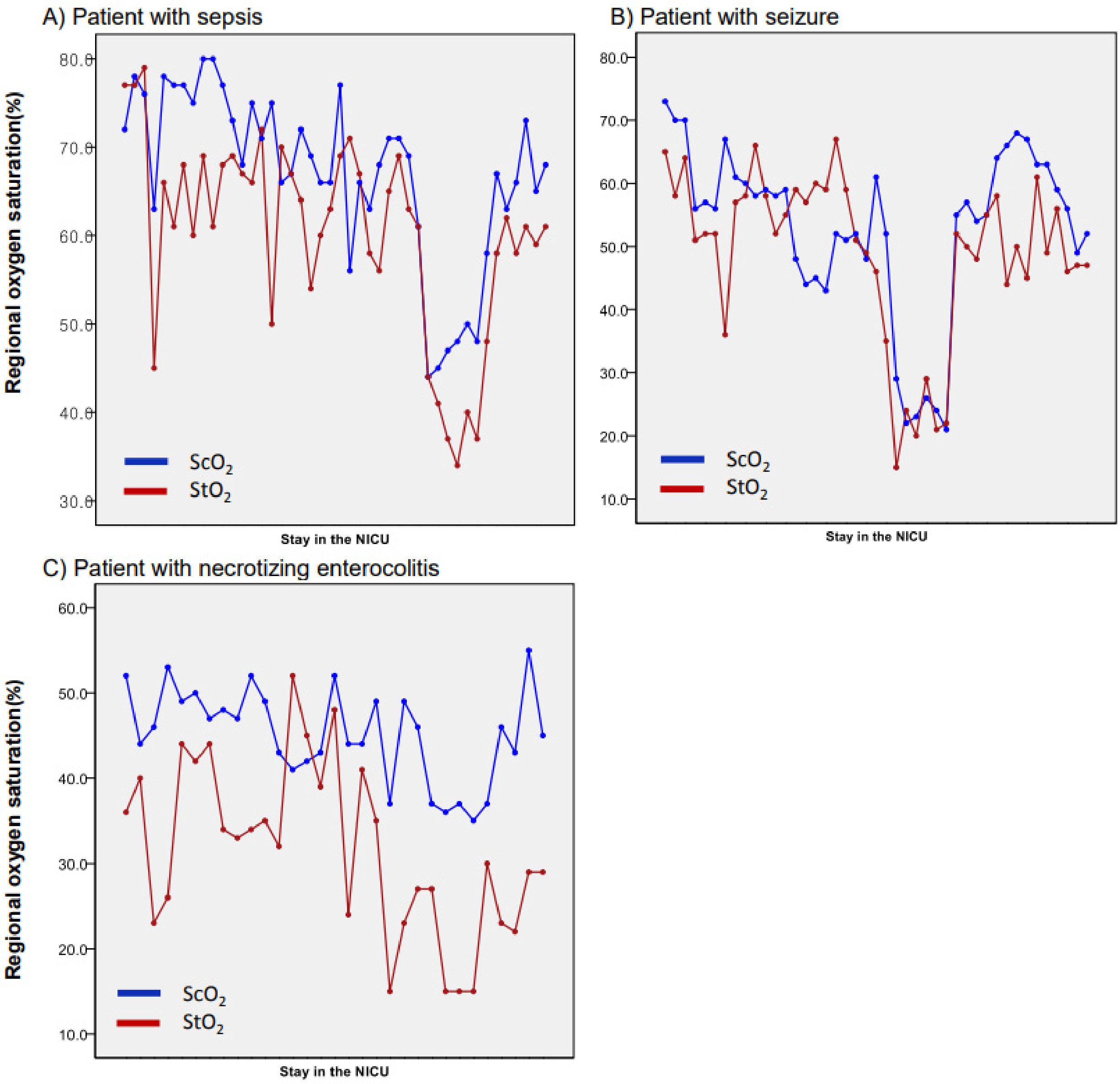

Supplement: Supplementary file 1 [file jcm-10-02455-s001.zip › jcm-1213857-supplementary.pdf]
